# Efficient Identification of Critical Residues Based Only on Protein Structure by Network Analysis

**DOI:** 10.1371/journal.pone.0000421

**Published:** 2007-05-09

**Authors:** Michael P. Cusack, Boris Thibert, Dale E. Bredesen, Gabriel del Rio

**Affiliations:** 1 Buck Institute for Age Research, Novato, California, United States of America; 2 Laboratoire de Modélisation et Calcul (LMC-IMAG), Grenoble, France; 3 University of California San Francisco, San Francisco, California, United States of America; 4 Universidad Nacional Autónoma de México/Instituto de Fisiología Celular, Departamento de Bioquímica, Mexico City, Mexico; Wellcome Trust Centre for Human Genetics, United Kingdom

## Abstract

Despite the increasing number of published protein structures, and the fact that each protein's function relies on its three-dimensional structure, there is limited access to automatic programs used for the identification of critical residues from the protein structure, compared with those based on protein sequence. Here we present a new algorithm based on network analysis applied exclusively on protein structures to identify critical residues. Our results show that this method identifies critical residues for protein function with high reliability and improves automatic sequence-based approaches and previous network-based approaches. The reliability of the method depends on the conformational diversity screened for the protein of interest. We have designed a web site to give access to this software at http://bis.ifc.unam.mx/jamming/. In summary, a new method is presented that relates critical residues for protein function with the most traversed residues in networks derived from protein structures. A unique feature of the method is the inclusion of the conformational diversity of proteins in the prediction, thus reproducing a basic feature of the structure/function relationship of proteins.

## Introduction

Deciphering protein function is one of the most active areas of research in biology involving both experimental and theoretical approaches [1], [2]. In that endeavor, identification of the critical residues for protein function constitutes a central area of research [3], [4], [5]. For instance, identification of critical residues in proteins is important for both protein function modulation (e.g., drug design [6]) and protein classification [4]. To this end, protein sequences constitute the first and most abundant source of data to infer protein function and hence most computational methods designed to identify critical residues are based on the analysis of protein sequences. However, protein function results from the three-dimensional structure adopted by the protein sequence and hence a protein's three-dimensional structure may be more appropriate to identify critical residues [7]. In that sense, a residue critical for a protein structure is as well critical for the protein function. Hence, referring to critical residues for protein function includes both types of residues: residues critical for protein structure and/or residues critical for its biological function (e.g., catalysis, binding).

With the increased capacity to determine the three-dimensional structures of proteins there has come an exponential growth in the public database of protein structures [8]. With this accumulation of data, new algorithms for predicting critical residues from protein's structure have emerged [9]–[11]. These new methods are especially important because protein structures sometimes are the only data source to predict critical residues, since at least 25% of the known proteins do not show significant sequence similarity with any other proteins [3], [12]. However, either few of these new algorithms based on protein structure are available [13]–[15] or these use sequence analysis as part of their approach [13]–[17]. Thus, in order to assist in the identification of critical residues considering this new trend on protein databases, it is important to develop structure-based methods that are at least as reliable as sequence-based methods and available for the scientific community to use.

We have recently described a method that uses only the protein structure to identify critical residues for protein function, based on the centrality measurement closeness centrality [11]. Our method is based on tracing shortest paths while traversing all the nodes in the net, so we refer to it as the Minimum Interacting Networks (MIN) method. Using a single structure for a given protein, MIN method detects critical residues with high sensitivity, and complements the predictions derived from sequence analysis approaches [11]. Alternatively, the centrality measurement called betweeness has been reported to be useful to identify critical residues for protein folding [18] or protein-protein interactions [10]. In any of these studies [10], [11], [18], the methods require the users to either provide the expected number of critical residues (however, most commonly there is no a priori knowledge to determine this number) or to use a statistical approach that depends on the amount of structural data available. In order for structure-based approaches to be used in a systematic fashion, these limitations need to be improved. In the current work, we report a highly specific method based on betweeness to identify critical residues, which sensitivity relays on the number and diversity of conformations provided (see Methods). In order to deal with the analysis of multiple protein structures, we describe an implementation that takes advantage of the multitask capacity embedded in Java™, that is, parallel processing and distributed computing [19] to give access to this software to the scientific community. We refer to this software as JAMMING (JAva-based Multi-threaded MIN-GUI).

First, we describe the method and its overall reliability to identifying known critical residues for protein function. Then, we show that including multiple structures of a protein of interest may be used to improve the reliability of our method and makes it more reliable than other automatic methods based on either protein sequence or protein structure. Our results indicate that JAMMING may be used to identify critical residues for protein function that are either critical for keeping the protein structure and/or for its biological function (e.g., catalysis, protein interactions).

## Results

### Algorithm

The underlying idea of JAMMING is that residues central for residue-residue contacts should be critical for protein function. Hence, our method is divided into three steps:

1. Building networks from protein structures.
2. Tracing the shortest path connecting every pair of residues in the network derived in step 1.
3. Find the residues with the largest dynamic connectivity (dk).

The results of each step (1 through 3) constitute the input for the next step. Additionally, in order to allow for JAMMING calculations to be executed at the same time either in multiple machines (JavaParty implementation) or through a web interface (Servlet implementation), the three steps were embedded in independent remote objects or Java™'s thread. Briefly, JavaParty [19] provides a framework that allows multi-threaded Java™ programs to be distributed on environments such as clusters based on the Remote Method Invocation (RMI) protocol using the Remote class that is a simple wrapper of the Thread class from the Java Standard Edition.

### Implementation

Here, we report the training of our method with the dynamic connectivity (dk) as a centrality measurement (see Methods). Briefly, dk estimates the frequency of a node to be traversed in connecting the whole network through shortest paths. Thus, a node with a large dk value is a highly traversed one. To determine the best protocol to build a network derived from a protein structure, we built 21 different types of networks using different criteria (see Table 1). In order to evaluate the best network, we used two sets of proteins (total 131 proteins, see Methods) and evaluated the sensitivity, specificity and error of the predictions based on the dk value to identify critical residues.

**Table 1:** Parameter sets used to generate networks from the protein structures in the T4L-TEM-HIVP and FSSP128 sets.

Dmin (Å)	Dmax (Å)	D_criterion_	Pairing	Error
0	3	Average	H	1.0
0	3	Average	All	1.0
0	3	Once	H	0.97
0	3	Once	Ch	0.96
0	3	Once	Ch+H	0.95
0	3	Once	All	0.71
0	4	Once	All	0.59
0	5	Average	Ch+H	1.0
0	5	Average	All	0.91
0	5	Once	H	0.85
0	5	Once	Ch	0.91
0	5	Once	Ch+H	0.83
0	5	Once	All	0.81
0	6	Once	All	0.68
0	7	Once	All	0.72
0	8	Once	All	0.68
0	10	Average	All	0.68
0	10	Once	Ch	0.89
0	15	Average	All	0.75
0	20	Average	Ch+H	0.88
5	20	Once	Ch	0.96

Dmin (Å): Minimum distance of separation in Angstroms (Å) between atoms or residues.

Dmax (Å): Maximum distance of separation (Å) between atoms or residues. D_criterion_: If once, residues were paired if at least one atom in each residue was within the specified D value. If average, the distance between the centers of mass of the residues was taken. Pairing: 4 rules for pairing residues were implemented: ch: the two residues are polar or charged and have complementary charges, ch+h: the two residues have complementary charges or both are hydrophobic, h: the two residues were hydrophobic and all: any two residues were paired. Error: An estimation of the reliability of the predictions of critical residues achieved using the corresponding network: the smaller the error the better the prediction (see Methods). The Error is indicated here only for the T4L-TEM-HIVP set.

**Figure 1:**
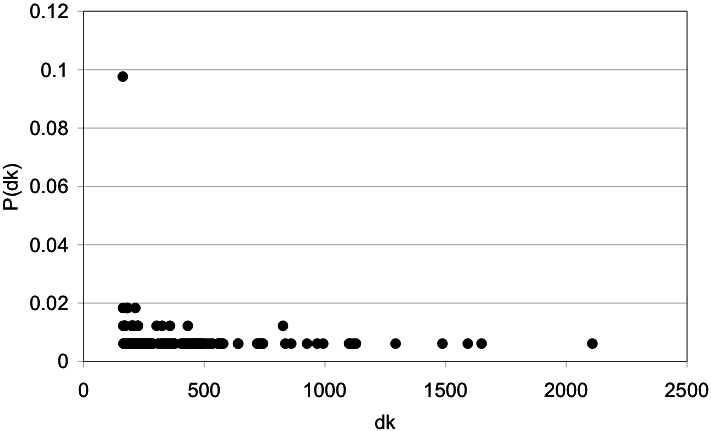
Distribution of the dynamic connectivity in a protein-derived network. The dynamic connectivity (df) observed in the T4 lysozyme protein (2LZM) is plotted against the probability of finding residues with such dk (P(dk)). In this type of plot, multiple residues are represented in a single dk value.

We plotted the frequency of the dk values in order to identify the most traversed residues from the networks derived from the protein three-dimensional structure. Such distribution showed a tendency to separate highly traversed residues (see Fig. 1 for an example) that we used to define an automatic procedure to isolate them (see Methods). In this case, the most traversed residues in the network are also the less frequent.

**Figure 2:**
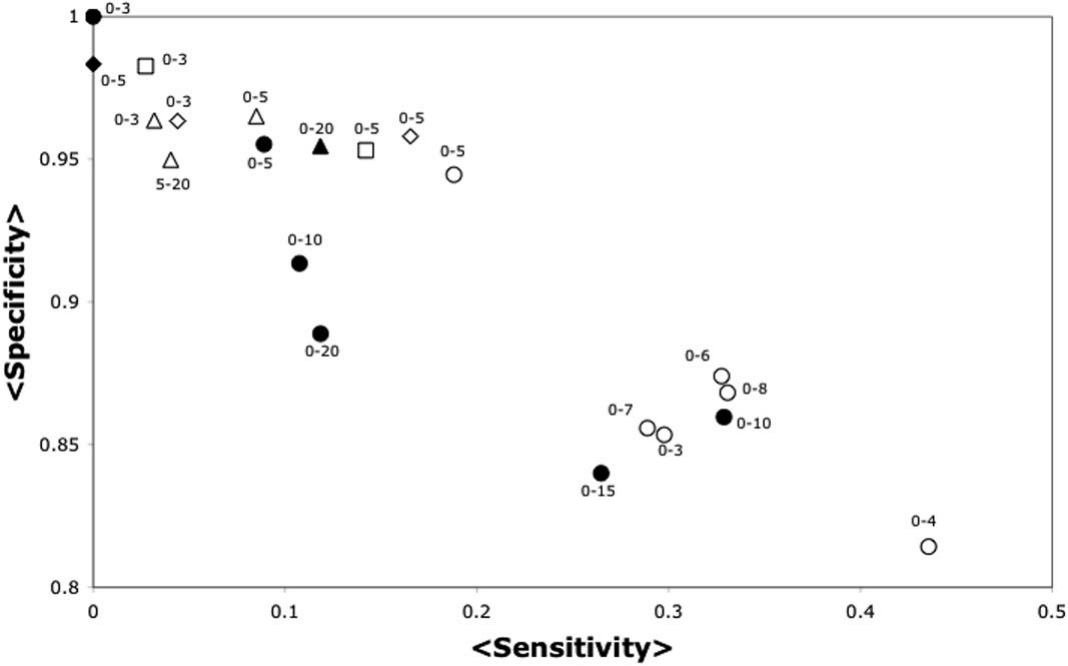
Sensitivity vs. Specificity of JAMMING in the T4L-TEM-HIVP set. The average sensitivity (axis labeled <Sensitivity>) and average specificity (axis labeled <Specificity>) over all the 3 proteins included in the T4L-TEM-HIVP set are presented for the predictions performed by JAMMING. Every point corresponds to a different network model according to Table 1: D_criterion_ = once is an empty symbol, D_criterion_ = average is a filled symbol, pairing hydrophobic residues is represented by a square, pairing complementary charged residues is represented by a triangle, pairing complementary charged residues and hydrophobic residues is represented by a rhombi and pairing every residue at the specified D_criterion_ is represented as a circle. The labels (e.g., 0-5) on each symbol in the graph, indicate the minimum and maximum distance used to establish the connections among the residues (see Methods and table 1). For instance, a 0-5 labeling an empty circle corresponds to the sensitivity and specificity obtained from networks derived from the T4L-TEM-HIVP set where any two residues were paired (symbol is a circle) if at least one atom between these residues (D_criterion_ = once, represented by an empty symbol) is within 0-5 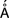 distance (label 0-5).

We are interested in identifying critical residues with the highest specificity and lowest error values, even if the sensitivity is low. As we will show below, the sensitivity of our method relays on the number of structures analyzed, so a method with high specificity in a single structure is desirable. We show for the T4L-TEM1-HIV1P set, that building networks pairing every residue (disregarding charge or any other criterion) rendered the best values for error and specificity (see Fig. 2 and Table 1). This trend was observed for all of the distances tested, but the complete data are only shown for the 0–3 Angstrom or 0–5 Angstrom distances. Additionally, networks built pairing residues 4 Å, 6 Å, 7 Å or 8 Å apart or closer rendered higher sensitivity values but lower specificity values than those at 5 Å.

**Figure 3:**
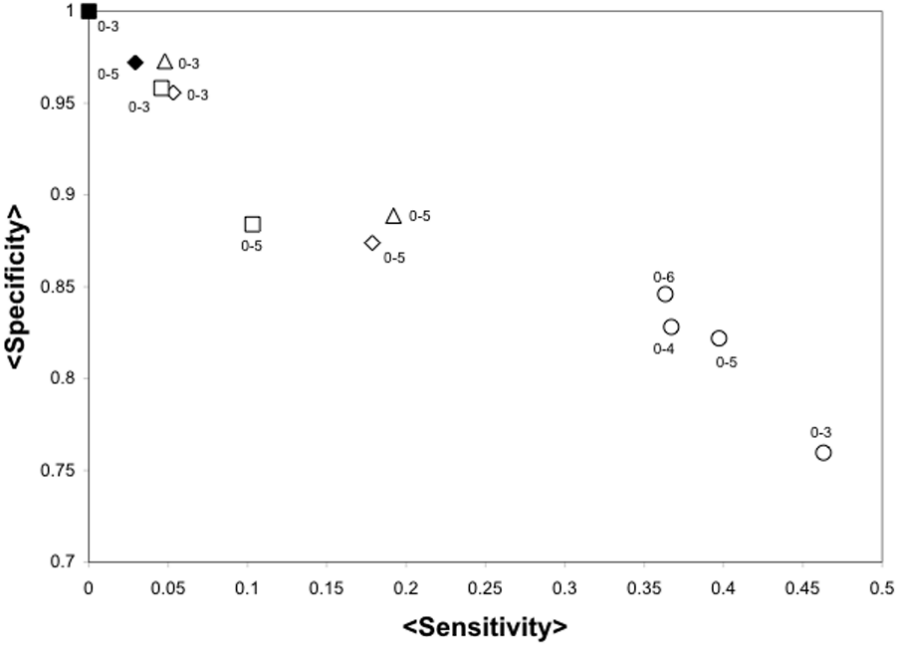
Sensitivity vs. Specificity of JAMMING in the FSSP128 dataset. The average sensitivity (axis labeled <Sensitivity>) and average specificity (axis labeled <Specificity>) over all the FSSP128 set are presented for the predictions performed by JAMMING. Every point corresponds to a different network model according to Table 1: D_criterion_ = once is an empty symbol, D_criterion_ = average is a filled symbol, pairing hydrophobic residues is represented by a square, pairing complementary charged residues is represented by a triangle, pairing complementary charged residues and hydrophobic residues is represented by a rhombus and pairing every residue at the specified D_criterion_ is represented as a circle. For simplicity, only the results for distance separations of 0-3 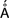, 0-4 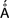, 0-5 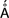 and 0-6 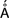 are presented. For instance, a 0-4 labeling an filled circle corresponds to the sensitivity and specificity obtained from networks derived from the FSSP128 set where any two residues were paired (symbol is a circle) if on average every atom between these residues (D_criterion_ = average, represented by a filled symbol) is within 0-4 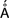 distance (label 0-4).

To extend these studies to a larger dataset, we used the FSSP128 set, where some of the critical residues are known. A similar set of proteins has been previously used to evaluate the predictive value of functionally important residue predictions [3]. We observed that networks built pairing residues at 5 Å apart or closer also rendered a high specificity in the predictions (see Fig. 3). The FSSP128 set includes as critical residues those in the SITE annotations of the PDB file. These annotations include mainly either residues observed in the structure to interact with a ligand or highly conserved residues, but do not include all possible critical residues. Alternatively, the T4L-TEM1-HIV1P set does include every critical residue for each protein. Thus, it is not surprising that the average sensitivity value for the FSSP128 set is larger than that observed for the T4L-TEM1-HIV1P set.

As with other centrality measurements [11], we noticed that the most traversed residues lay on both the protein's core and surface (see Fig. S1 and S2), but with a trend to be on the protein's core. Specifically, in the FSSP128 set 85.8% of the predicted critical residues are buried within the protein core and 14.2% are exposed; these percentages are obtained by considering as part of the protein's core those residues with a relative surface area of 50% or less (see Fig. S1 for details). This trend is complementary to sequence-conserved residues as we noted previously for another centrality measurements [11]. Furthermore, in the T4L-HIV-TEM1 set we observed again that most of the predicted critical resides by JAMMING have a role on structure (85%) but some have a catalytic role (15%) (see Table S1).

### Improving sensitivity by including multiple protein structures

**Figure 4:**
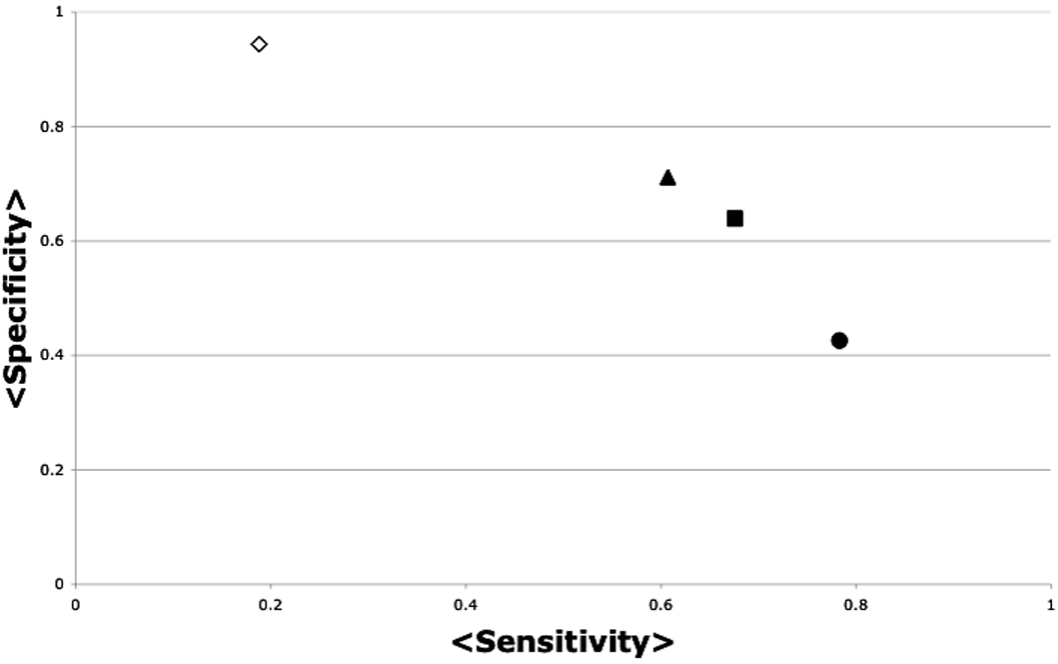
Sensitivity vs. Specificity of JAMMING using multiple protein structures. The average sensitivity (axis labeled <Sensitivity>) and average specificity (axis labeled <Specificity>) over all the 3 proteins included in the T4L-TEM-HIVP set and its normal mode perturbed models are presented. The triangles represent the data including 2 low-frequency modes (modes 10 and 11, see Methods section); the squares represent the 2 lowest-frequency modes (modes 30 and 31, see Methods); the circles represent the data including 5 normal modes. Each mode was used to generate 10 perturbed models from the initial PDB structure (see Methods section), so the average values in this plot represent 44 (triangles), 44 (squares) and 165 (circles) protein structures. For comparison, the sensitivity and specificity obtained using a single protein structure for each protein in this set is represented in an empty rhomb.

Considering that proteins are not static molecules, it is accepted that an ensemble of protein structures accomplishes protein function [20]. Thus, in order to identify most of the critical residues for protein function (to improve the sensibility of our method) is important to include in the analysis several protein structures. Normal mode analysis is a powerful method for predicting as much as half of the possible movements of proteins with only two normal modes [21]. Thus, we would expect that using multiple conformations that represent most of the conformational diversity of a protein could improve the sensibility of our approach. In that case, our goal is to produce a method capable of reproducing at least the reliability of automatic sequence-based approaches. For example, we have previously evaluated two automatic methods based on sequence analysis, and biased them providing the correct number of critical residues to be predicted. In that case, these methods achieved an average error of 44% (sensibility 76% and specificity 67%) using the T4L-TEM1-HIV1P set [11]. However, in the most common scenario where no information is available about the number of critical residues for a protein, following the common assumption that critical residues are the most conserved ones [22], the average error value increases for this same set to 49.6% (sensibility 53.3% and specificity 83.3%). As we have shown above, considering a single protein structure does not render this level of reliability (see Fig. 2). In Fig. 4 we show for this same set of proteins that using multiple structures for a given protein may improve the reliability of our method to an average error value of 48.4% (sensitivity 67.6% and specificity 63.9%). Another important implication from our results is that the number of structures included in the analysis does not determine the reliability of the predictions, but the conformational diversity sampled by the structures (i.e., using the lowest-frequency modes).

### Web site deployment and software distribution

In order to provide public access to JAMMING, we developed a web site using the Java™'s servlet technology. The servlet (master program) may render requests to a group of registered machines (workers). Yet, the current servlet implementation only deploys the requests to a single computer. Alternatively, we have made available a command-line version of our program through our web site that is ready to be used in a cluster environment as well as a GUI to be run on a single computer. As noted above, sometimes it is important to include multiple structures for a given protein to have a good reliability in the predictions. So, it is important to keep in mind that running multiple analyses in a single CPU may not be feasible, depending on the specifications of the hardware, so it is usually convenient to have the chance to use multiple processors.

## Discussion

The accumulated information on protein structures and community efforts such as the structural genomics initiative [23] may lead to a better understanding of protein functions beyond what sequence analysis is rendering nowadays [1]. One important step towards that goal is the identification of critical residues for protein function. Here we present the implementation of an algorithm written in the Java™ programming language aimed to detect critical residues from protein structures. Our implementation, dubbed JAMMING, is based on the identification of the most traversed residues from protein structure-derived networks.

We trained JAMMING with different types of protein structure-derived networks and found that pairing every residue at 5 Å apart or closer rendered the most reliable predictions on average. Our results show that pairing residues that display complementary physicochemical properties does not improve the identification of critical residues, indicating that the optimization of our network model does not require residue-type discrimination. It is important to note that our previous work using closeness centrality to identify critical residues from protein structures [11] employed the same criteria for building networks as the one described here. We suggested then [11] that our approach for building networks offered an improved reliability (sensitivity 70%, specificity 70%) over that described by Amitai and colleagues [9] (sensitivity 40%, specificity 10%) that uses the same centrality measurement, closeness centrality, to identify critical residues.

Previous studies have indicated that critical residues tend to be buried in the protein's core [24], while few critical residues are on the protein's surface, such as active site residues. Our results are consistent with this notion: most critical residues detected by JAMMING (∼85%) lay on the protein's core. To explain our results in terms of network connectivity, lets assume that proteins adopt a three-dimensional structure where all residues are regularly packed; in this case, it is likely that the most traversed residues will always be in the protein's core. However, we observe that the most traversed residues lie on both the protein's core and protein's surface (see Figs. S1 and S2 and Table S1). This suggests that the three-dimensional structures of proteins do not necessarily feature regular packing. In other words, if proteins will have regularly distributed connections on the three-dimensional space (such as equally dense polymers), the most central residues will always be on the protein's core. In agreement with this observation, it has previously been reported that protein structure-derived networks present a non-regular distribution referred to as the small-world phenomena [18]. We do not know yet, however, whether the three-dimensional localization of the most traversed residues depend on the small-world character observed in the packing of protein structures or any other topological property.

Other approaches to identify critical residues with different basis than ours [3], [4], [5], [9], [13], [16], [17] may be combined to improve the predictive capacity of these methods. For instance, we have shown that closeness-centrality could be as accurate and complementary to sequence-based detection of critical residues, provided that the correct number of critical residues is previously known [11]. However, the exact number of critical residues for a given protein is not commonly known, thus limiting the usefulness of these approaches.

On the other hand, JAMMING is shown to improve the reliability of automatic sequence-based approaches by considering multiple normal mode perturbed models of a given protein. In that sense, JAMMING may be especially useful in cases where there are a limited number of sequences to perform a sequence analysis.

When using our approach to analyze other protein structures, we noticed that some structures only hold as central residues those in the protein's core (data not shown), but including multiple structures of a protein always render critical residues on the protein's surface as well. So, as a general approach, the researcher may choose first to identify critical residues based exclusively on a protein structure provided that the specificity of the method is quite good in that condition, and depending on the goal of the researcher it may be useful to run JAMMING using multiple protein structures.

So far, we have described the reliability of JAMMING in average terms. Now, we will describe some specific predictions obtained with it (see Table S1). Our goal is dual: i) to highlight predictions not attainable from the sequence and ii) to describe the usefulness of our method to identify residues involved in binding (i.e., active sites, protein-protein interfaces).

The Arg145 residue in the bacteriophage T4 lysozyme was identified by our method to be critical for the protein function, yet this residue is not conserved [24]. From the three-dimensional structure of the bacteriophage T4 lysozyme it has been observed that Arg145 forms part of a buried salt bridge with the catalytic residue Glu11 [24], suggesting a role in stabilizing the conformation of this catalytic residue. Interestingly, mutations on Arg145 do not indicate a stringent requirement for this salt bridge, since Arg145 can be replaced without deleterious effects for the protein function with uncharged residues [24]. On the other hand, most substitutions of this residue abolished the activity of the lysozyme indicating a critical role for the function of the enzyme [24]. Thus, sequence analysis revealed that Arg145 can be replaced by non-conservative amino acids during the evolution of this enzyme (Arg145 is aligned with Cys, Val, Gln, Glu, Lys, Ile, Asp, Pro and Thr, based on the alignment reported for the lysozyme at the Dali server [25]), and yet our method identified this residue as critical for the protein function. These data indicates that in the bacteriophage T4 lysozyme, position 145 plays an important role for the protein function and an Arginine residue is adequate for it, yet in other lysozymes different residues may accomplish that function. In this example we can appreciate how our method complements sequence analysis.

Now we will analyze a couple of residues involved in binding sites. As we have shown [11], conserved residues tend to be on the protein surface, thus sequence analysis in combination with structure analysis may render a good prediction of binding sites [14]. However, having the ability to identify binding sites based exclusively on protein structure may be important when few homologue sequences are available for a given protein, such as in the case of viral proteins or proteins with new folds, for instance. The proteins analyzed here all have many homologue sequences, but these serve to exemplify the usefulness of our approach in the identification of binding sites based only on protein structure.

One of these residues is Thr71 in the TEM-1 beta-lactamase. This residue is not conserved among the class A beta-lactamases (enzymes capable of hydrolyzing beta-lactam antibiotics mostly encoded in plasmid in Gram(+) and Gram(−) bacteria), but it is conserved among the TEM1 beta-lactamases (the most common plasmid-mediated beta-lactamase). This conservation pattern suggests a possible role in specificity. Random mutagenesis at this position supports this notion [26]. This level of prediction can be achieved mainly because of the large number and diversity of protein sequences available for this protein family. On the other hand, our method used only one three-dimensional structure of the TEM1 beta-lactamase to identify this surface-exposed residue as critical. That is, JAMMING may be useful in identifying residues critical for the distinctive function of proteins even when few homologue sequences are known.

Another residue involved in binding is Leu24 from the HIV-1 protease. This is a conserved residue and it is located in the interface of the protease homodimer. Interestingly, our method predicted this residue as critical for the protein function considering only the monomer of the protease. In agreement, mutagenesis of Leu24 showed small tolerance to substitutions at this position [27]. Thus, for the Thr71 in the TEM-1 beta-lactamase and Leu24 in the HIV-1 protease, JAMMING used only the structural information of a protein without any knowledge about its interactions, indicating that the protein structure holds information about its function that is identifiable by our procedure.

The ability to identify critical residues for protein structure/function represents a basic tool for many important areas in bioinformatics, including protein structure prediction, protein function design or functional classification, among others. In many of these areas, a program capable to be run in a systematic fashion is desirable. JAMMING is the first available software of its class that may be used to complement other approaches or be used where others are limited (*e.g*., proteins with known structure but limited sequence information).

In summary, JAMMING is a multi-threaded Java™ implementation of a new approach aimed at detecting critical residues from protein structures. Our method is suitable to be executed in multiple machines or on a single one. JAMMING is available at http://bis.ifc.unam.mx/jamming/.

## Methods

### Systems

Three Linux boxes (RedHat 7.2) with 2 Pentium III processors each were used to run the described calculations. These boxes were configured as a cluster with the Rocks framework (http://www.rocksclusters.org). Additionally, 1 Linux and 1 MacOS×computers were used to test our implementation in a heterogeneous computer system (see Implementation below). This was distributed using the JavaParty framework (http://www.ipd.uka.de/JavaParty/).

All of the procedures described in this work were coded in the Java™ programming language (http://java.sun.com). Two types of implementations were developed in this work. The first one was developed to test JAMMING when multiple protein structures are being analyzed. This program may be executed on a cluster of computers using the JavaParty framework. Also, this program may be executed in a single computer without the JavaParty framework. In both cases we used Java's Threads allowing to switch from one to another without major modification on the code (see below) The second implementation was a Java™'s servlet that provides a web interface to access JAMMING for single protein structure analysis. The WebMol Applet (http://www.cmpharm.ucsf.edu/∼walther/webmol.html) was adapted to graphically display the results of the predictions by the servlet. Only protein atoms are considered in this version of the program.

### Data

Two protein data sets were used to test our method: the T4L-TEM-HIVP set and the FSSP128 set. Briefly, the T4L-HIV-TEM1 set includes three enzymes that have been extensively characterized structurally and functionally (TEM1 beta-lactamase (PDB code 1BTL), HIV-1 protease (PDB code 1HIV) and T4 lysozyme (PDB code 2LZM)). The FSSP128 set includes 128 other proteins for which there is a partial annotation regarding the critical residues (single chained FSSP protein entries with SITE annotations). The 128 PDB names used in this set are: 1a3c, 1a6q, 1a7j, 1aac, 1ac5, 1ah7, 1ak1, 1ako, 1amj, 1an8, 1apq, 1arv, 1atg, 1auz, 1ayl, 1ayx, 1az9, 1b64, 1bag, 1bdb, 1bea, 1bfd, 1bia, 1bif, 1bix, 1bk0, 1bli, 1bn5, 1bor, 1boy, 1bp1, 1bqk, 1brt, 1btl, 1c25, 1ca1, 1cby, 1cex, 1cfb, 1chc, 1chd, 1csh, 1ctn, 1ctt, 1cvl, 1dmr, 1drw, 1dxy, 1ecl, 1eh2, 1emn, 1esl, 1eut, 1far, 1fnc, 1gca, 1htn, 1hyt, 1iba, 1ido, 1iow, 1iyu, 1kcw, 1kpf, 1lam, 1lay, 1lbu, 1lgr, 1lml, 1lox, 1mfs, 1mla, 1mrp, 1mup, 1nif, 1opc, 1pda, 1pdc, 1pfo, 1phd, 1phm, 1pii, 1pkp, 1poa, 1poc, 1rfs, 1rie, 1rkd, 1rlw, 1skf, 1snc, 1sra, 1thx, 1uch, 1uox, 1ush, 1whi, 1wod, 1xbd, 1xpa, 1ytw, 2abk, 2adr, 2af8, 2cba, 2cmd, 2dkb, 2dri, 2fha, 2fua, 2liv, 2mcm, 2mnr, 2rn2, 2sas, 2vil, 3dfr, 3dni, 3ebx, 3gcb, 3pte, 3ssi, 3tgl, 4enl, 4icb, 4pah, 5eat and 7rsa.

### Building networks from protein structures

Networks were derived from protein structures by a distance criterion. That is, two residues were considered neighbors and consequently paired in the network if they were within a given distance from each other. Two different distance criteria were used: a) two residues were considered neighbors if any of their atoms were within the specified distance, and b) two residues were considered neighbors if their centers of mass were within the specified distance. Additionally, four filters were used to define neighbors, that is, given that the distance criteria were satisfied, the residues being paired were considered neighbors if: i) the two residues were polar or charged and had complementary charges (Asp, Glu, Asn, Gln, Tyr, Phe were paired with Arg, Lys, His, Asn, Gln), ii) the two residues had complementary charges or both were hydrophobic (Asp, Glu, Asn, Gln, Tyr, Phe, Ala, Leu, Ile, Val, Trp, Ser, Thr, Cys, Met were paired with Arg, Lys, His, Asn, Gln, Ala, Leu, Ile, Val, Trp, Ser, Thr, Cys, Met), iii) the two residues were hydrophobic (Ala, Leu, Ile, Val, Trp, Ser, Thr, Cys, Met were paired with Ala, Leu, Ile, Val, Trp, Ser, Thr, Cys, Met), or iv) any two residues were paired. Therefore, different types of networks were built specifying 7 separation distances, 2 distance criteria and four pairing criteria (see Table 1 for a list of different parameters used to build networks). The networks built had amino acid residues as nodes and their interactions as links. Links were labeled with identical weight and were bidirectional.

### Finding the central residues from protein-derived networks

In order to find the central residues from protein structure-derived networks, every pair of residues were connected through a shortest path. We used the Dijkstra's algorithm for tracing such shortest paths [28]. By counting how many times every residue is traversed in connecting every pair of residues in the graph we define the node's dynamic connectivity, dk. Betweeness and dk are related: betweeness = dk/(N(N-1)), where N is the number of nodes in the graph. We referred to dynamic connectivity of a residue, as opposed to a static connectivity named the node's degree (i.e., number of direct neighbors to a node). By looking at the frequency of dk values in protein structures (dk vs. the probability of such dynamic connectivity, P(dk); P(dk) is obtained by counting the number of times a given dk value is present in the graph divided by N) we define the most traversed residues as those with the largest dynamic connectivity values having the same smallest P(dk) value in the distribution. For instance, in figure 1 we can graphically identify the most traversed residues as those presenting the largest dk values on the right-lower corner. Note that a single dk value includes multiple residues with the same dk value.

### Estimating the reliability of the predictions

Two measurements, sensitivity and specificity, were used to account for the reliability of the method tested, as described in [11]. To estimate these parameters, we first count the number of experimentally determined critical residues (E), the number of non-critical residues (NE = (protein sequence's length)-E), the number of total predicted critical residues (P), the number of truly predicted critical residues (TP) and the number of false predicted critical residues (FP = P-TP). Hence, Sensitivity is defined as Se = TP/E and Specificity as Sp = (NE-FP)/NE. E values were those experimentally determined and annotated as a SITE for the T4L-TEM-HIVP set and FSSP128 set respectively. From these parameters we evaluated the error (how far form perfection is the method) associated with a predictive method [11]: 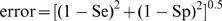



### Normal mode perturbed models generation

The *ElNemo* web service to compute the normal modes of the T4L-TEM-HIVP set was used [21]. Briefly, 25 normal modes were calculated, starting from the non-trivial mode 7, and for each 11 PDB models were obtained. These include the reference structure and 10 models were generated using a range of amplitude values (−100 to 100, with increments of 20). The atoms in the protein were grouped by the ‘rotation-translation-block’ approximation. The cutoff distance for elastic interactions was set to 8 Angstroms.

## Supporting Information

Table S1 Predicted critical residues and their annotated function in the T4L-HIV-TEM1 set.
(0.12 MB DOC)

Figure S1 Relationship between the surface area and dk centrality measurement for the FSSP128 set of proteins
(0.25 MB TIF)

Figure S2 Structural location of the most traversed residues
(0.31 MB TIF)

## References

[1] Eisenberg D, Marcotte EM, Xenarios I, Yeates TO (2000). Protein function in the post-genomic era. Nature,.

[2] Thornton JM, Todd AE, Milburn D, Borkakoti N, Orengo CA (2000). From structure to function: approaches and limitations. Nat Struct Biol,.

[3] del Sol Mesa A, Pazos F, Valencia A (2003). Automatic methods for predicting functionally important residues. J Mol Biol,.

[4] Devos D, Valencia A (2000). Practical limits of function prediction. Proteins,.

[5] Elcock AH (2001). Prediction of functionally important residues based solely on the computed energetics of protein structure. J Mol Biol,.

[6] Mattos C, Ringe D (1996). Locating and characterizing binding sites on proteins. Nat Biotechnol,.

[7] Benkovic SJ, Hammes-Schiffer S (2003). A perspective on enzyme catalysis. Science,.

[8] Berman HM, Battistuz T, Bhat TN, Bluhm WF, Bourne PE (2002). The protein data bank Acta Crystallogr D Biol Crystallogr,.

[9] Amitai G, Shemesh A, Sitbon E, Shklar M, Netanely D (2004). Network analysis of protein structures identifies functional residues. J. Mol. Biol..

[10] del Sol A, O'Meara P (2005). Small-world network approach to identify key residues in protein-protein interaction.. Proteins,.

[11] Thibert B, Bredesen DE, del Rio G (2005). Improved prediction of critical residues for protein function based on network and phylogenetic analyses.. BMC Bioinformatics.

[12] Sadreyev RI, Grishin NV (2006). Exploring dynamics of protein structure determination and homology-based prediction to estimate the number of superfamilies and folds.. BMC Structural Biology.

[13] Dundas J, Ouyang Z, Tseng J, Binkowski A, Turpaz Y (2006). CASTp: computed atlas of surface topography of proteins with structural and topographical mapping of functionally annotated residues. Nucleic Acids. Res..

[14] Glaser F, Pupko T, Paz I, Bell RE, Bechor-Shental D (2003). ConSurf: identification of functional regions in proteins by surface-mapping of phylogenetic information. Bioinformatics,.

[15] Pal D, Eisenberg D (2005). Inference of protein function from protein structure. Structure.

[16] George RA, Spriggs RV, Bartlett GJ, Gutteridge A, MacArthur MW (2005). Effective function annotation through catalytic residue conservation. Proc. Natl. Acad. Sci. USA.

[17] Pazos F, Sternberg MJ (2004). Automated prediction of protein function and detection of functional sites from structure. Proc. Natl. Acad. Sci. USA.

[18] Vendruscolo M, Dokholyan NV, Paci E, Karplus M (2002). Small-world view of the amino acids that play a key role in protein folding.. Phys Rev E Stat Nonlin Soft Matter Phys..

[19] Phillippsen M, M Z (1997). JavaParty-transparent remote objects in Java Concurrency: Practice and Experience,.

[20] Mittermaier A, Lay LE (2006). New tools provide new insights in NMR studies of protein dynamics.. Science.

[21] Suhre K, Sanejouand Y-H (2004). ElNémo: a normal mode web server for protein movement analysis and the generation of templates for molecular replacement.. Nucl.Acids Res..

[22] Poteete AR, Renell D, Bouvier SE (1992). Functional significance of conserved amino acid residues.. Proteins: structure, function and genetics.

[23] Christendat D, Yee A, Dharamsi A, Kluger Y, Savchenko A (2000). Structural proteomics of an archaeon. Nat Struct Biol,.

[24] Rennell D, Bouvier SE, Hardy LW, Poteete RA (1991). Systematic mutation of bacteriophage T4 lysozyme. J Mol Biol.

[25] Holm L, Ouzounis C, Sander C, Tuparev G, Vriend G (1992). A database of protein structure families with common folding motifs.”.. Protein Science.

[26] Huang W, Petrosino J, Hirsch M, Shenkin PS, Palzkill T (1996). Amino acid sequence determinants of β-lactamase structure and activity.. J. Mol. Biol..

[27] Loeb DD, Swanstrom R, Everitt L, Manchester M, Stamper S (1989). Complete mutagenesis of the HIV-1 protease.. Nature.

[28] Weiss MA, Hartman S (1999). Graph algorithms.. Data structures & algorithm analysis in Java™.

