# Supplementary material for: Efficient Identification of Critical Residues Based Only on Protein Structure by Network Analysis
Source: PLoS One. 2007 May 9;2(5):e421. doi: 10.1371/journal.pone.0000421 (PMC1855080; doi:10.1371/journal.pone.0000421)
Supplement: Table S1 — Predicted critical residues and their annotated function in the T4L-HIV-TEM1 set. (0.12 MB DOC) [file pone.0000421.s001.doc]

**Table 1. *Predicted critical residues and their annotated function in the T4L-HIV-TEM1 set*.**

| **Residue (**[**1HIV**](http://bis.ifc.unam.mx/jamming/SupplementaryData/1hiv_webmol.html)**)** | **Function** | **Residue (**[**1BTL**](http://bis.ifc.unam.mx/jamming/SupplementaryData/1btl_webmol.html)**)** | **Function** | **Residue (**[**2LZM**](http://bis.ifc.unam.mx/jamming/SupplementaryData/2lzm_webmol.html)**)** | **Function** |
| --- | --- | --- | --- | --- | --- |
| ASN83 | [Structure](http://www.ncbi.nlm.nih.gov/entrez/query.fcgi?db=pubmed&cmd=Retrieve&dopt=AbstractPlus&list_uids=2666861&query_hl=6&itool=pubmed_docsum) | MET211 | [Structure](http://www.ncbi.nlm.nih.gov/entrez/query.fcgi?db=pubmed&cmd=Retrieve&dopt=AbstractPlus&list_uids=8637002&query_hl=4&itool=pubmed_docsum) | GLU11 | [Catalytic](http://www.ncbi.nlm.nih.gov/entrez/query.fcgi?db=pubmed&cmd=Retrieve&dopt=AbstractPlus&list_uids=1942069&query_hl=2&itool=pubmed_docsum) |
| LEU33 | [Structure](http://www.ncbi.nlm.nih.gov/entrez/query.fcgi?db=pubmed&cmd=Retrieve&dopt=AbstractPlus&list_uids=2666861&query_hl=6&itool=pubmed_docsum) | LYS73 | [Catalytic](http://www.ncbi.nlm.nih.gov/entrez/query.fcgi?db=pubmed&cmd=Retrieve&dopt=AbstractPlus&list_uids=8637002&query_hl=4&itool=pubmed_docsum) | PHE104 | [Structure](http://www.ncbi.nlm.nih.gov/entrez/query.fcgi?db=pubmed&cmd=Retrieve&dopt=AbstractPlus&list_uids=1942069&query_hl=2&itool=pubmed_docsum) |
| ILE47 | [Structure](http://www.ncbi.nlm.nih.gov/entrez/query.fcgi?db=pubmed&cmd=Retrieve&dopt=AbstractPlus&list_uids=2666861&query_hl=6&itool=pubmed_docsum) | LEU207 | [Not critical](http://www.ncbi.nlm.nih.gov/entrez/query.fcgi?db=pubmed&cmd=Retrieve&dopt=AbstractPlus&list_uids=8637002&query_hl=4&itool=pubmed_docsum) | LEU7 | [Structure](http://www.ncbi.nlm.nih.gov/entrez/query.fcgi?db=pubmed&cmd=Retrieve&dopt=AbstractPlus&list_uids=1942069&query_hl=2&itool=pubmed_docsum) |
| LEU24 | [Structure](http://www.ncbi.nlm.nih.gov/entrez/query.fcgi?db=pubmed&cmd=Retrieve&dopt=AbstractPlus&list_uids=2666861&query_hl=6&itool=pubmed_docsum) | ASN132 | [Substrate binding](http://www.ncbi.nlm.nih.gov/entrez/query.fcgi?db=pubmed&cmd=Retrieve&dopt=AbstractPlus&list_uids=8637002&query_hl=4&itool=pubmed_docsum) | ILE100 | [Structure](http://www.ncbi.nlm.nih.gov/entrez/query.fcgi?db=pubmed&cmd=Retrieve&dopt=AbstractPlus&list_uids=1942069&query_hl=2&itool=pubmed_docsum) |
| LEU23 | [Structure](http://www.ncbi.nlm.nih.gov/entrez/query.fcgi?db=pubmed&cmd=Retrieve&dopt=AbstractPlus&list_uids=2666861&query_hl=6&itool=pubmed_docsum) | SER70 | [Catalytic](http://www.ncbi.nlm.nih.gov/entrez/query.fcgi?db=pubmed&cmd=Retrieve&dopt=AbstractPlus&list_uids=8637002&query_hl=4&itool=pubmed_docsum) | PHE67 | [Structure](http://www.ncbi.nlm.nih.gov/entrez/query.fcgi?db=pubmed&cmd=Retrieve&dopt=AbstractPlus&list_uids=1942069&query_hl=2&itool=pubmed_docsum) |
| VAL32 | [Structure](http://www.ncbi.nlm.nih.gov/entrez/query.fcgi?db=pubmed&cmd=Retrieve&dopt=AbstractPlus&list_uids=2666861&query_hl=6&itool=pubmed_docsum) | ARG244 | [Substrate binding](http://www.ncbi.nlm.nih.gov/entrez/query.fcgi?db=pubmed&cmd=Retrieve&dopt=AbstractPlus&list_uids=8637002&query_hl=4&itool=pubmed_docsum) | ILE3 | [Structure](http://www.ncbi.nlm.nih.gov/entrez/query.fcgi?db=pubmed&cmd=Retrieve&dopt=AbstractPlus&list_uids=1942069&query_hl=2&itool=pubmed_docsum) |
| ILE85 | [Structure](http://www.ncbi.nlm.nih.gov/entrez/query.fcgi?db=pubmed&cmd=Retrieve&dopt=AbstractPlus&list_uids=2666861&query_hl=6&itool=pubmed_docsum) | TRP210 | [Structure](http://www.ncbi.nlm.nih.gov/entrez/query.fcgi?db=pubmed&cmd=Retrieve&dopt=AbstractPlus&list_uids=8637002&query_hl=4&itool=pubmed_docsum) | LEU99 | [Structure](http://www.ncbi.nlm.nih.gov/entrez/query.fcgi?db=pubmed&cmd=Retrieve&dopt=AbstractPlus&list_uids=1942069&query_hl=2&itool=pubmed_docsum) |
| ARG57 | [Structure](http://www.ncbi.nlm.nih.gov/entrez/query.fcgi?db=pubmed&cmd=Retrieve&dopt=AbstractPlus&list_uids=2666861&query_hl=6&itool=pubmed_docsum) | LEU139 | [Not critical](http://www.ncbi.nlm.nih.gov/entrez/query.fcgi?db=pubmed&cmd=Retrieve&dopt=AbstractPlus&list_uids=8637002&query_hl=4&itool=pubmed_docsum) | ILE29 | [Structure](http://www.ncbi.nlm.nih.gov/entrez/query.fcgi?db=pubmed&cmd=Retrieve&dopt=AbstractPlus&list_uids=1942069&query_hl=2&itool=pubmed_docsum) |
| ILE15 | [Structure](http://www.ncbi.nlm.nih.gov/entrez/query.fcgi?db=pubmed&cmd=Retrieve&dopt=AbstractPlus&list_uids=2666861&query_hl=6&itool=pubmed_docsum) | PHE72 | [Not critical](http://www.ncbi.nlm.nih.gov/entrez/query.fcgi?db=pubmed&cmd=Retrieve&dopt=AbstractPlus&list_uids=8637002&query_hl=4&itool=pubmed_docsum) | ILE27 | [Structure](http://www.ncbi.nlm.nih.gov/entrez/query.fcgi?db=pubmed&cmd=Retrieve&dopt=AbstractPlus&list_uids=1942069&query_hl=2&itool=pubmed_docsum) |
| ILE13 | [Structure](http://www.ncbi.nlm.nih.gov/entrez/query.fcgi?db=pubmed&cmd=Retrieve&dopt=AbstractPlus&list_uids=2666861&query_hl=6&itool=pubmed_docsum) | TYR264 | [Not critical](http://www.ncbi.nlm.nih.gov/entrez/query.fcgi?db=pubmed&cmd=Retrieve&dopt=AbstractPlus&list_uids=8637002&query_hl=4&itool=pubmed_docsum) | MET102 | [Structure](http://www.ncbi.nlm.nih.gov/entrez/query.fcgi?db=pubmed&cmd=Retrieve&dopt=AbstractPlus&list_uids=1942069&query_hl=2&itool=pubmed_docsum) |
| ASP30 | [Catalytic](http://www.ncbi.nlm.nih.gov/entrez/query.fcgi?db=pubmed&cmd=Retrieve&dopt=AbstractPlus&list_uids=2666861&query_hl=6&itool=pubmed_docsum) | PHE66 | [Structure](http://www.ncbi.nlm.nih.gov/entrez/query.fcgi?db=pubmed&cmd=Retrieve&dopt=AbstractPlus&list_uids=8637002&query_hl=4&itool=pubmed_docsum) | TRP138 | [Structure](http://www.ncbi.nlm.nih.gov/entrez/query.fcgi?db=pubmed&cmd=Retrieve&dopt=AbstractPlus&list_uids=1942069&query_hl=2&itool=pubmed_docsum) |
|  |  | ILE247 | [Not critical](http://www.ncbi.nlm.nih.gov/entrez/query.fcgi?db=pubmed&cmd=Retrieve&dopt=AbstractPlus&list_uids=8637002&query_hl=4&itool=pubmed_docsum) | ILE17 | [Structure](http://www.ncbi.nlm.nih.gov/entrez/query.fcgi?db=pubmed&cmd=Retrieve&dopt=AbstractPlus&list_uids=1942069&query_hl=2&itool=pubmed_docsum) |
|  |  | TYR46 | [Structure](http://www.ncbi.nlm.nih.gov/entrez/query.fcgi?db=pubmed&cmd=Retrieve&dopt=AbstractPlus&list_uids=8637002&query_hl=4&itool=pubmed_docsum) | TYR18 | [Structure](http://www.ncbi.nlm.nih.gov/entrez/query.fcgi?db=pubmed&cmd=Retrieve&dopt=AbstractPlus&list_uids=1942069&query_hl=2&itool=pubmed_docsum) |
|  |  | PHE230 | [Not critical](http://www.ncbi.nlm.nih.gov/entrez/query.fcgi?db=pubmed&cmd=Retrieve&dopt=AbstractPlus&list_uids=8637002&query_hl=4&itool=pubmed_docsum) | ARG145 | [Catalytic/Structure](http://www.ncbi.nlm.nih.gov/entrez/query.fcgi?db=pubmed&cmd=Retrieve&dopt=AbstractPlus&list_uids=1942069&query_hl=2&itool=pubmed_docsum) |
|  |  | VAL74 | [Not critical](http://www.ncbi.nlm.nih.gov/entrez/query.fcgi?db=pubmed&cmd=Retrieve&dopt=AbstractPlus&list_uids=8637002&query_hl=4&itool=pubmed_docsum) |  |  |
|  |  | THR71 | [Substrate binding](http://www.ncbi.nlm.nih.gov/entrez/query.fcgi?db=pubmed&cmd=Retrieve&dopt=AbstractPlus&list_uids=8637002&query_hl=4&itool=pubmed_docsum) |  |  |
|  |  | LEU75 | [Not critical](http://www.ncbi.nlm.nih.gov/entrez/query.fcgi?db=pubmed&cmd=Retrieve&dopt=AbstractPlus&list_uids=8637002&query_hl=4&itool=pubmed_docsum) |  |  |
|  |  | VAL44 | [Not critical](http://www.ncbi.nlm.nih.gov/entrez/query.fcgi?db=pubmed&cmd=Retrieve&dopt=AbstractPlus&list_uids=8637002&query_hl=4&itool=pubmed_docsum) |  |  |
|  |  | GLU48 | [Not critical](http://www.ncbi.nlm.nih.gov/entrez/query.fcgi?db=pubmed&cmd=Retrieve&dopt=AbstractPlus&list_uids=8637002&query_hl=4&itool=pubmed_docsum) |  |  |
|  |  | LEU190 | [Not critical](http://www.ncbi.nlm.nih.gov/entrez/query.fcgi?db=pubmed&cmd=Retrieve&dopt=AbstractPlus&list_uids=8637002&query_hl=4&itool=pubmed_docsum) |  |  |
|  |  | MET186 | [Not critical](http://www.ncbi.nlm.nih.gov/entrez/query.fcgi?db=pubmed&cmd=Retrieve&dopt=AbstractPlus&list_uids=8637002&query_hl=4&itool=pubmed_docsum) |  |  |
|  |  | MET69 | [Not critical](http://www.ncbi.nlm.nih.gov/entrez/query.fcgi?db=pubmed&cmd=Retrieve&dopt=AbstractPlus&list_uids=8637002&query_hl=4&itool=pubmed_docsum) |  |  |
|  |  | MET68 | [Not critical](http://www.ncbi.nlm.nih.gov/entrez/query.fcgi?db=pubmed&cmd=Retrieve&dopt=AbstractPlus&list_uids=8637002&query_hl=4&itool=pubmed_docsum) |  |  |
|  |  | SER235 | [Substrate binding](http://www.ncbi.nlm.nih.gov/entrez/query.fcgi?db=pubmed&cmd=Retrieve&dopt=AbstractPlus&list_uids=8637002&query_hl=4&itool=pubmed_docsum) |  |  |

Critical residues as predicted by JAMMING in the unsupervised version are presented with their known function for the three well characterized enzymes, HIV-1 proteaase (1HIV), beta-lactamase (1BTL) and T4 lysozyme (2LZM). The function "Structure" refer to those positions that do not tolerate substitutions and no evidence is known for a catalytic or substrate binding function. "Catalytic" or "Substrate binding" functions corresponds to those positions that do not tolerate substitutions and that there is biochemical and/or structural evidence regarding catalysis or substrate's binding. The "Not critical" function corresponds to those positiosn predicted as critical but that tolerate substitutions whithout affecting the enzyme activity under the experimetal conditions tested. The table allows you to see the three dimensional localization of these residues by clicking on the Residue table header. The references from which the known function were obtained are accesible by clicking on the annotated function.
