# Supplementary figures and images for: Efficient Identification of Critical Residues Based Only on Protein Structure by Network Analysis
Source: PLoS One. 2007 May 9;2(5):e421. doi: 10.1371/journal.pone.0000421 (PMC1855080; doi:10.1371/journal.pone.0000421)

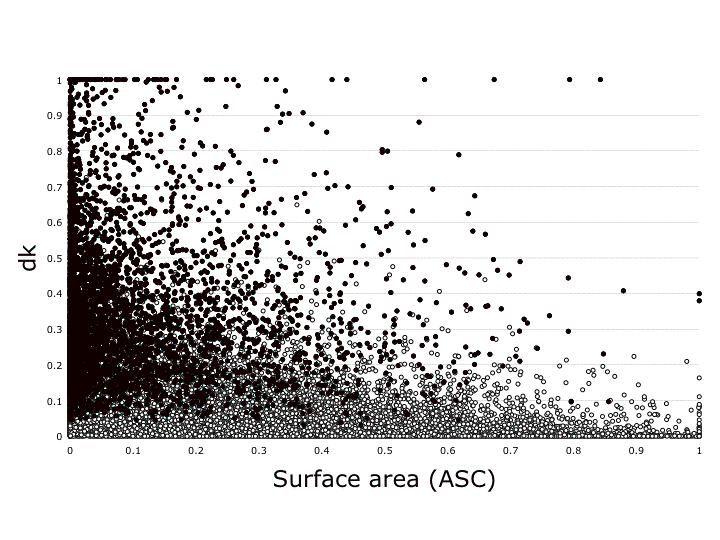

Supplement: Figure S1 — Relationship between the surface area and dk centrality measurement for the FSSP128 set of proteins (0.25 MB TIF) [file pone.0000421.s002.tif]

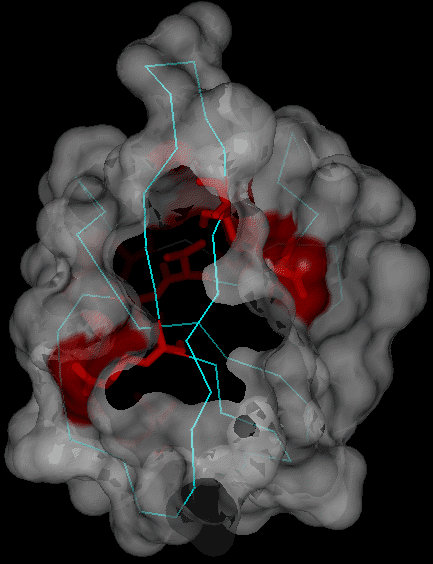

Supplement: Figure S2 — Structural location of the most traversed residues (0.31 MB TIF) [file pone.0000421.s003.tif]
